# Synthesis and Degradation of the Phytohormone Indole-3-Acetic Acid by the Versatile Bacterium *Paraburkholderia xenovorans* LB400 and Its Growth Promotion of *Nicotiana tabacum* Plant

**DOI:** 10.3390/plants13243533

**Published:** 2024-12-18

**Authors:** Paulina Vega-Celedón, Diyanira Castillo-Novales, Guillermo Bravo, Franco Cárdenas, María José Romero-Silva, Michael Seeger

**Affiliations:** 1 Molecular Microbiology and Environmental Biotechnology Laboratory, Department of Chemistry, Universidad Técnica Federico Santa María, Avenida España 1680, Valparaíso 2390123, Chile; diyaniracastillonovales@gmail.com (D.C.-N.); bravoc.guillermo@gmail.com (G.B.); mjromerosilva@gmail.com (M.J.R.-S.); 2 Center of Biotechnology “Daniel Alkalay Lowitt”, Universidad Técnica Federico Santa María, General Bari 699, Valparaíso 2390136, Chile; 3 Escuela de Agronomía, Facultad de Ciencias Agronómicas y de los Alimentos, Pontificia Universidad Católica de Valparaíso, San Francisco s/n La Palma, Quillota 2260000, Chile; 4 Millennium Nucleus Bioproducts, Genomics and Environmental Microbiology (BioGEM), Avenida España 1680, Valparaíso 2390123, Chile

**Keywords:** indole-3-acetic acid (IAA), IAA synthesis, IAA degradation, *Paraburkholderia xenovorans* LB400, plant growth-promoting bacteria (PGPB)

## Abstract

Plant growth-promoting bacteria (PGPB) play a role in stimulating plant growth through mechanisms such as the synthesis of the phytohormone indole-3-acetic acid (IAA). The aims of this study were the characterization of IAA synthesis and degradation by the model aromatic-degrading bacterium *Paraburkholderia xenovorans* LB400, and its growth promotion of the *Nicotiana tabacum* plant. Strain LB400 was able to synthesize IAA (measured by HPLC) during growth in the presence of tryptophan and at least one additional carbon source; synthesis of anthranilic acid was also observed. RT-PCR analysis indicates that under these conditions, strain LB400 expressed the *ipdC* gene, which encodes indole-3-pyruvate decarboxylase, suggesting that IAA biosynthesis proceeds through the indole-3-pyruvate pathway. In addition, strain LB400 degraded IAA and grew on IAA as a sole carbon and energy source. Strain LB400 expressed the *iacC* and *catA* genes, which encode the α subunit of the aromatic-ring-hydroxylating dioxygenase in the IAA catabolic pathway and the catechol 1,2-dioxygenase, respectively, which may suggest a peripheral IAA pathway leading to the central catechol pathway. Notably, *P. xenovorans* LB400 promoted the growth of tobacco seedlings, increasing the number and the length of the roots. In conclusion, this study indicates that the versatile bacterium *P. xenovorans* LB400 is a PGPB.

## 1. Introduction

Plant growth-promoting bacteria (PGPB) are beneficial for plants, contributing to plant growth, health, and protection against phytopathogens via the synthesis of phytohormones, the increase of nutrient availability (e.g., nitrogen fixation, phosphorus solubilization, siderophore synthesis) and the control of plant pathogens [1,2]. Extensively studied in recent years, PGPB belong to various genera, including *Acetobacter*, *Acinetobacter*, *Alcaligenes*, *Arthrobacter*, *Azoarcus*, *Azospirillum*, *Azotobacter*, *Bacillus*, *Beijerinckia*, *Burkholderia*, *Derxia*, *Enterobacter*, *Gluconacetobacter*, *Herbaspirillum*, *Klebsiella*, *Ochrobactrum*, *Pantoea*, *Paraburkholderia*, *Pseudomonas*, *Rhodococcus*, *Serratia*, *Stenotrophomonas*, and *Zoogloea* [1,2,3].

Indole-3-acetic acid (IAA) is a phytohormone that serves as the primary endogenous auxin in higher plants, influencing various physiological processes such as cell division, elongation, tissue differentiation, phototropism, gravitropism, and defensive responses [4,5]. Plants but also bacteria, fungi, and algae are able to synthesize IAA, impacting plant growth and development [6,7]. Notably, IAA production is a distinguishing feature of both PGPB and phytopathogenic bacteria [8,9]. Five tryptophan-dependent IAA biosynthetic pathways have been reported in bacteria; the indole-3-pyruvate (IPyA) and indole-3-acetamide (IAM) pathways are the most important and widely distributed [10,11,12]. The IPyA pathway is prevalent in PGPB, while the IAM pathway is primarily associated with phytopathogenic bacteria. Anthranilic acid (AA) acts as a metabolic intermediate in tryptophan synthesis and degradation, which typically does not accumulate [13]. It has been reported that AA promotes the growth of maize plants [14], participating in phytohormone synthesis pathways (e.g., IAA), and exhibiting bacteriostatic activity against the phytopathogen *Streptomyces* sp. B-9-1 [15]. IAA-degrading bacteria are also associated with plants [4]. IAA is an aromatic compound that may be used as a carbon and nitrogen source for bacterial growth. Complete aerobic IAA degradation has been reported in bacterial genera *Bradyrhizobium*, *Pseudomonas*, *Burkholderia*, *Paraburkholderia*, *Rhodococcus*, *Sphingomonas*, *Caballeronia*, *Enterobacter*, and *Acinetobacter* [16,17,18]. The mineralization of IAA or its transformation into a biologically inactive molecule allows the bacterium to manipulate physiological activities related to IAA levels in plants [16]. The aerobic catabolic pathway that transforms IAA into catechol is codified by the *iac* gene cluster, which was first described in *Pseudomonas putida* 1290 [16,18].

Diverse *Paraburkholderia* species are plant-associated bacteria and rhizobia that present a multifaceted role in improving plant performance such as enhancement of nutrient mobilization and plant growth, tolerance to abiotic and biotic stress, reduction of oxidative stress, improvement of root architecture, and production of secondary metabolites [19,20]. *Paraburkholderia xenovorans* LB400 (previously named *Burkholderia xenovorans* LB400) is a non-pathogenic aerobic soil bacterium, with a large genome size of 9.73 Mbp, which is a model for the degradation of aromatic compounds [21]. Strain LB400 degrades an unusually wide range of aromatic compounds including polychlorobiphenyls (PCBs) and is capable of modifying flavonoids [21,22,23,24,25,26,27,28,29,30,31]. Other *P. xenovorans* strains have been isolated from coffee, corn, and tomato plant rhizospheres and human blood [32,33,34,35]. Caballero-Mellado et al. [35] reported that *P. xenovorans* strains TCo-382 and TCo-213 from tomato plants have the potential to improve plant growth, due to their 1-aminocyclopropane-1-carboxylic acid (ACC) deaminase activity, which lowers ethylene levels in plants. However, these strains are unable to degrade PCBs [32,35]. In this study, the aims were the characterization of the IAA synthesis and degradation by *P. xenovorans* LB400, and its growth promotion of the *Nicotiana tabacum* plant.

## 2. Results and Discussion

### 2.1. IAA Synthesis

#### 2.1.1. *P. xenovorans* LB400 Possesses Genes for IAA Biosynthesis

**Figure 1:**
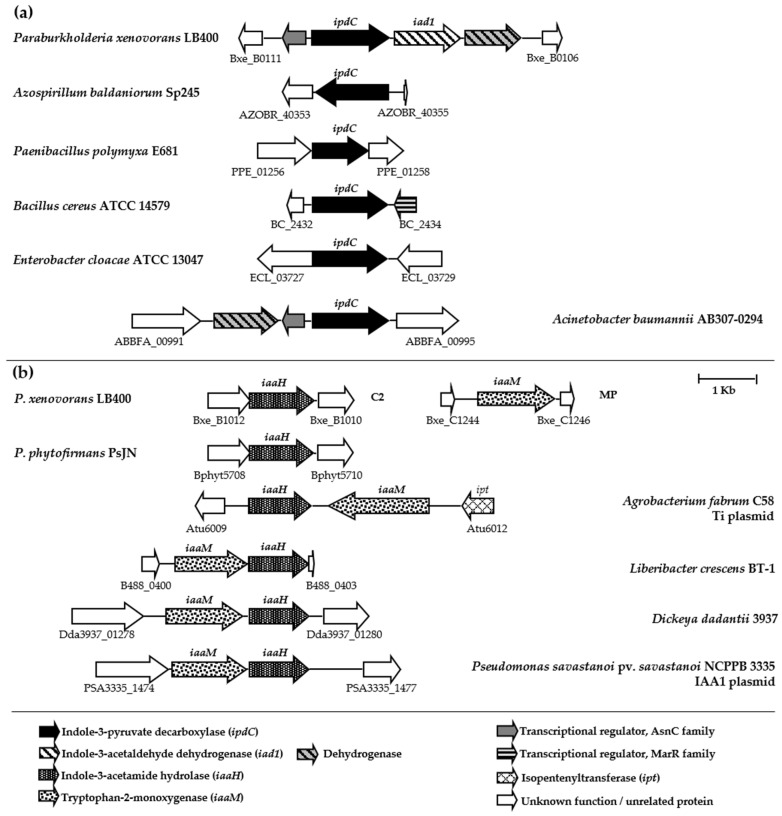
Organization of genes in the IPyA and IAM IAA anabolic pathways in *P. xenovorans* LB400 and other bacteria. (**a**) Organization of the *ipdC* and *iad1* genes of the indole-3-pyruvate (IPyA) pathway. (**b**) Organization of *iaaH* and *iaaM* genes of the indole-3-acetamide (IAM) pathway. Gene sizes and intergenic regions are accurately represented to scale. C2 denotes the minor chromosome, and MP represents the megaplasmid.

Bioinformatic analysis of the *P. xenovorans* LB400 genome unveiled the presence of genes encoding two distinct pathways for IAA synthesis from tryptophan: the IPyA and IAM routes (Table 1). The IPyA pathway is primarily associated with plant growth-promoting bacteria (PGPB), while the IAM pathway is observed mainly in phytopathogenic bacteria [10,11]. The IPyA route involves three enzymatic steps. Initially, the precursor tryptophan undergoes transamination into IPyA via an aminotransferase. Thirty-nine genes encoding aminotransferases have been reported in the LB400 genome [21]. In the rate-limiting step, IPyA is decarboxylated to form indole-3-acetaldehyde (IAAld) by the indole-3-pyruvate decarboxylase (IpdC), encoded by the *ipdC* gene. Subsequently, IAAld is oxidized to IAA by the indole-3-acetaldehyde dehydrogenase, encoded by the *iad1* gene. In strain LB400, the *ipdC* (Bxe_B0109) and *iad1* (Bxe_B0108) genes are clustered on the minor chromosome (C2). Bioinformatic analysis suggests that the *ipdC* and *iad1* genes form a transcriptional unit (Figure 1a). The products of the *ipdC* and *iad1* genes in strain LB400 exhibit ≥ 37% amino acid sequence identity with IpdC and Iad1 proteins from the bacterium *Pantoea agglomerans* 299R and the fungus *Ustilago maydis* FB1, respectively [36,37] (Table 1).

The *ipdC* gene has been studied in bacteria such as *Azospirillum baldaniorum*, *Paenibacillus polymyxa*, *Bacillus cereus*, *Enterobacter cloacae*, and *Acinetobacter baumannii* [38,39,40,41,42,43,44,45,46]. Recently, indole-3-acetaldehyde dehydrogenase (AldA) from *Ps. syringae* pv. *tomato* DC3000 has been described [47]. The indole-3-acetaldehyde dehydrogenase of the strain DC3000 showed 37% amino acid sequence identity with the Iad1 protein from strain LB400. The organization of the *ipdC* and *iad1* genes in strain LB400 is interesting and differs from other bacteria possessing the IPyA pathway, since these genes are clustered on its genome (Figure 1a).

In the LB400 genome, adjacent to the *ipdC* and *iad1* genes are located genes encoding an FMN-dependent dehydrogenase (Bxe_B0107), and a transcriptional regulator from the AsnC family (Bxe_B0110) that is also present in *Acinetobacter baumannii* strain AB307-0294 (Figure 1a). In *A. baumannii* AB307-0294, the gene encoding a transcriptional regulator from the AsnC family is positioned between the *ipdC* gene and a dehydrogenase gene (Figure 1a). AsnC proteins are part of the Lrp global transcriptional regulators [48], known to regulate the expression of amino acid anabolic and catabolic pathways and related processes [49]. The AsnC protein from *P. xenovorans* LB400 displays the characteristic helix-turn-helix (HTH) motif for DNA binding in the N-terminal and the effector-binding domain αβ-sandwich in the C-terminal. A potential Lrp-type regulatory binding sequence, CACTACATTGTAGTC, was identified upstream of the *ipdC* gene in strain LB400, exhibiting high identity (75%) with the consensus Lrp-type regulator binding sequence YAGHAWATTWTDCTR [50].

The IAM pathway for IAA synthesis involves two key enzymatic steps. Initially, tryptophan undergoes oxidation into IAM by tryptophan monooxygenase, which is encoded by the *iaaM* gene. Subsequently, IAM is hydrolyzed into IAA and ammonia by IAM hydrolase, which is encoded by the *iaaH* gene. In *P. xenovorans* LB400, the *iaa* genes are distributed across different replicons. Specifically, the *iaaM* gene (Bxe_C1245) is located on the megaplasmid (MP), while the *iaaH* gene (Bxe_B1011) is located on C2 (Figure 1b). The *iaaM* and *iaaH* gene products in strain LB400 exhibit ≥ 38% sequence identity with IaaM and IaaH proteins from *Agrobacterium fabrum* C58 [51] (Table 1). The gene organization of the *iaa* genes differs among bacteria (Figure 1b). In the PGPB *P. phytofirmans* PsJN, only the *iaaH* gene is present. In the phytopathogens *Ps. savastanoi* pv. *savastanoi* NCPPB 3335 (plasmid IAA1) [52], *Liberibacter crescens* BT-1 [53], and *Dickeya dadantii* 3937 [54], both *iaaM* and *iaaH* genes are clustered with the same orientation. In the phytopathogens *Agrobacterium fabrum* C58 and *A. tumefaciens* strains [51,55], the *iaaM* and *iaaH* genes are clustered in the T-DNA region of the Ti plasmid, with opposite orientations; the *ipt* gene is located next to these genes, which encodes an isopentenyltransferase involved in cytokinin production, thereby enhancing its virulence [56]. The *ipt* gene is absent in the *P. xenovorans* LB400 genome.

#### 2.1.2. Synthesis of IAA by *P. xenovorans* LB400

**Figure 2:**
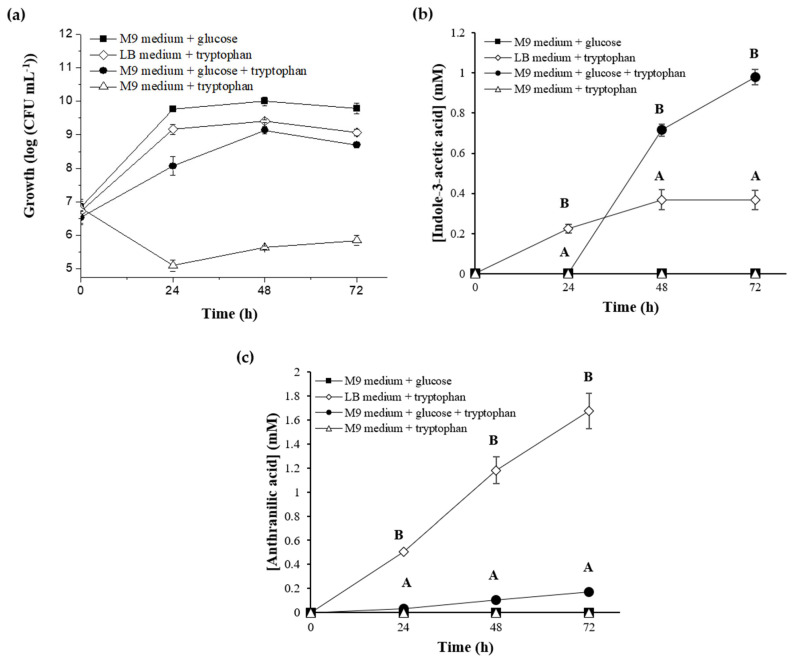
Growth and indole-3-acetic acid (IAA)/anthranilic acid (AA) biosynthesis by *P. xenovorans* LB400. (**a**) Growth in the presence of tryptophan. Cells were cultivated in different media (M9 or LB) with glucose (30 mM) and/or tryptophan (10 mM). (**b**) IAA biosynthesis. (**c**) AA biosynthesis. Each value represents an average ± SD of three independent experiments. IAA/AA production were not detected in M9 medium with tryptophan or glucose as sole carbon and energy sources. Significant differences for each time point (24 h, 48 h, 72 h) between the conditions of IAA/AA biosynthesis were analyzed by one-way ANOVA followed by the LSD Fisher test. Means with different letters indicate significant differences (*p* ≤ 0.05).

In this study, *P. xenovorans* LB400 cells were cultivated in different media and reverse-phase liquid chromatography was used to evaluate and quantify IAA synthesis (Figure 2). Recently, Ghitti et al. [57] described the auxin production of *P. xenovorans* LB400 using the non-specific Salkowski method. The Salkowski method detects IAA but also IPyA and indole-3-acetamide, intermediate compounds in IAA production, and other auxins such as indole-3-butyric acid [10,57,58].

*P. xenovorans* LB400 exhibited IAA synthesis in M9 medium with glucose supplemented with tryptophan, as well as in LB medium supplemented with tryptophan (Figure 2). In contrast, IAA production was not observed when strain LB400 was grown in M9 medium with sole glucose. In M9 medium with tryptophan as the sole carbon and energy source, LB400 growth and IAA production was not observed. However, the addition of glucose to M9 medium with tryptophan favored IAA production by strain LB400. Glucose supplementation enhances IAA production in *Rhizobium*, *Bacillus*, and *Streptomyces* strains grown with tryptophan [59,60,61,62,63]. IAA biosynthesis in strain LB400 was particularly prominent during the stationary growth phase (Figure 2a,b). Strain LB400 achieved its maximum IAA concentration (0.98 mM) after 72 h growth in M9 medium with both glucose and tryptophan (Figure 2a,b). Similar to strain LB400, other PGPB such as *Burkholderia pyrrocinia* JK-SH007, *Serratia plymuthica* UBCF_13, *Azospirillum baldoniarum* strains CBG 497 and Sp245, *Pantoea agglomerans* PVM, *Arthrobacter globiformis* A3, and *Rhizobium* spp. from nodule root of *Roystonea regia* and *Cajanus cajan* grown with tryptophan also showed higher IAA synthesis during the stationary phase [59,60,62,64,65,66,67,68,69,70]. By comparing the IAA production of strain LB400 with the IAA-producing bacterium *Pseudomonas protegens* CHA0 [71], which has the tryptophan side chain oxidase (TSO) IAA synthesis pathway, *P. xenovorans* strain LB400 exhibited higher IAA levels in all rich media (Luria-Bertani (LB), Yeast-Malt (YM), and King B (KB)) plus tryptophan (5 mM) than *Ps. protegens* strain CHA0 (Table 2). The YM medium plus tryptophan showed the highest IAA production for both strains, which has been also reported in *Serratia* [70]. The IAA production by *P. xenovorans* strain LB400 surpassed that of strain CHA0 reported in previous studies [71]. *Pantoea agglomerans* PVM, *Enterobacter hormaechei* VR2, and *Bacillus aryabhattai* MG9, and a *Rhizobium* spp. strain isolated from a nodule root of *Cajanus cajan* displayed high IAA production [57,60,65].

#### 2.1.3. Synthesis of Anthranilic Acid by *P. xenovorans* LB400

The observation of the accumulation of a secondary compound during the growth of strain LB400 in the presence of tryptophan and at least one additional carbon source led to the identification of anthranilic acid (AA) through HPLC analysis. The synthesis of AA by LB400 cells was observed in both LB medium with tryptophan and M9 medium with glucose and supplemented with tryptophan (Figure 2c), showing higher AA accumulation in LB medium with tryptophan. In LB medium supplemented with tryptophan, strain LB400 achieved an AA concentration of 1.67 mM after 72 h. Interestingly, LB400 cells in M9 medium with glucose and supplemented with tryptophan exhibited the highest IAA production with lower AA accumulation. In contrast, in LB medium supplemented with tryptophan, the highest AA production with lower IAA production was observed (Figure 2b,c). Since LB medium contains a higher tryptophan concentration than the M9 minimal medium due to the amino acid content of its composition provided by yeast extract and tryptone [72], the results suggest that in the presence of a high tryptophan concentration, strain LB400 favors AA accumulation, probably by activating the catabolism of tryptophan into AA. The comparison of AA production by *P. xenovorans* LB400 and the model PGPB *Ps. protegens* CHA0 in three rich media containing tryptophan (5 mM) indicated higher synthesis of AA by strain LB400 than by strain CHA0 under these conditions (Table 2). Specifically, *P. xenovorans* strain LB400 exhibited the highest AA synthesis (2.01 mM) in KB medium, which is the medium that has the highest concentration of digested peptides provided by peptone [73]. For strain CHA0, the highest AA (0.095 mM) levels were observed in YM medium. Notably, the AA synthesis by *P. xenovorans* LB400 was much higher than by *Ps. protegens* CHA0. This study highlights that *P. xenovorans* LB400 in the presence of tryptophan synthesized both IAA and AA. Simultaneous IAA and AA synthesis has been also described in *Azospirillum brasilense* CBG 497 and *Bradyrhizobium japonicum* BJBV-05 grown in LB medium with tryptophan [64,74].

*P. xenovorans* strain LB400 possesses the *kynABU* genes (Bxe_A0733, Bxe_A0734, and Bxe_B0735, respectively) clustered on C1, encoding the enzymes tryptophan 2,3-dioxygenase (tryptophan to formyl kynurenine), kynurenine formamidase (formyl kynurenine to kynurenine), and kynureninase (kynurenine to anthranilic acid) involved in the three-step degradation of tryptophan into AA [75]. In *Escherichia coli*, a high tryptophan concentration leads to the repression of *trp* operon transcription by the tryptophan-activated repressor protein TrpR, resulting in the attenuation of its transduction and the accumulation of its precursor AA [76,77]. In this study, we searched in the LB400 genome for genes involved in tryptophan synthesis. Strain LB400 possesses the *trpE* (Bxe_A0461), *trpD1* (Bxe_A0460), *trpD2* (Bxe_A0459), and *trpC1* genes (Bxe_A0458) grouped in the *trpED1D2C1* cluster on C1. The *trpE* gene encodes anthranilate synthase, which catalyzes the transformation of chorismate into anthranilate [78]. Additionally, the *trpC2* (Bxe_B2882), *trpB* (Bxe_B2881), and *trpA* genes (Bxe_B2879) are clustered on C2. A putative *trpR* gene (Bxe_A1353) encoding a TrpR-type transcriptional repressor is located on C1. In strain LB400, a high concentration of tryptophan may repress the expression of the *trpED1D2C* genes, supporting the accumulation of AA. Interestingly, the chemical synthesis of AA from petroleum compounds is a costly process involving high temperature and pressure [13], converting the biological production of AA into an attractive alternative strategy. Future investigations should explore potential biotechnological applications of AA production by *P. xenovorans* strain LB400.

#### 2.1.4. *P. xenovorans* Expressed the *ipdC* Gene but Not the *iaaH* Gene

**Figure 3:**
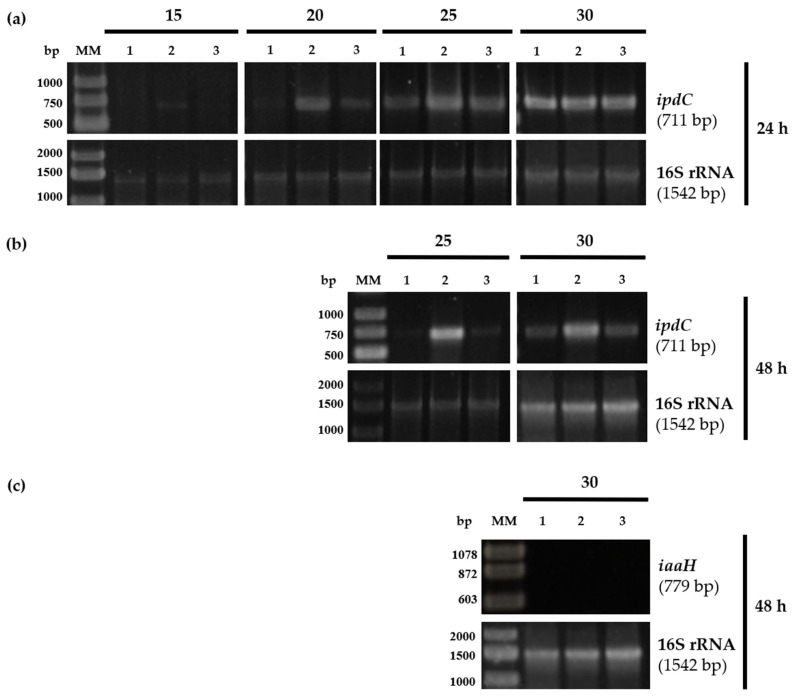
Expression of the *ipdC* and *iaaH* genes in *P. xenovorans* LB400 grown with different carbon sources. (**a**) RT-PCR (15, 20, 25, and 30 amplification cycles) of *ipdC* gene after 24 h growth. (**b**) RT-PCR (25 and 30 amplification cycles) of *ipdC* gene after 48 h growth. (**c**) RT-PCR (30 amplification cycles) of *iaaH* gene after 48 h growth. RNA samples were purified from LB400 cells harvested at 24 and 48 h. The 16S rRNA gene was selected as a reference gene. MM, molecular markers; 1, cells grown in M9 medium supplemented with glucose (30 mM); 2, cells grown in M9 medium supplemented with glucose (30 mM) and tryptophan (10 mM); 3, cells grown in LB medium supplemented with tryptophan (10 mM).

Transcriptional analysis of key metabolic genes of strain LB400 was conducted, to establish a connection between IAA biosynthesis and the anabolic genes. The expression in strain LB400 of the *ipdC* gene, which encodes the enzyme indole-3-pyruvate decarboxylase (IPyA pathway), and the *iaaH* gene, responsible for indole-3-acetamide hydrolase (IAM pathway), were examined. The *ipdC* gene was transcribed in all tested conditions (Figure 3a,b). However, a notable increase in *ipdC* gene expression was observed in *P. xenovorans* LB400 cells after 24 and 48 h of growth in M9 medium supplemented with glucose and tryptophan compared with M9 medium supplemented with glucose. These results suggest that in M9 medium supplemented with glucose, tryptophan enhances the expression of the *ipdC* gene in strain LB400.

The *ipdC* gene is upregulated in the presence of tryptophan in *Bacillus thuringiensis* RZ2MS9, *Enterobacter cloacae* UW5, *E. xiangfangensis* BWH6, *E. asburiae* STY10, *Pseudomonas putida* GR12-2, and *Azospirillum baldoniarum* Sp7, indicating its significance in IAA biosynthesis [8,41,47,79,80,81,82]. As tryptophan is prevalent in root exudates, plant tissues, and microbial debris, the observed upregulation of the *ipdC* gene in response to tryptophan may be involved in the adaptation to these specific environments. In *Pantoea agglomerans* 299R, the *ipdC* gene expression is plant-inducible, suggesting its involvement in plant–microbe interactions [69,83]. In strain LB400, the highest expression of the *ipdC* gene was observed in the stationary growth phase, correlating with higher IAA levels, which has also been described in *Enterobacter cloacae* UW5, *Pseudomonas putida* GR12-2, and *Serratia plymuthica* A153 [8,41,69,84]. In promoters of the *ipdC* gene from *A. brasilense* Sp245 and plant genes, *cis* elements with the consensus sequence TGTCNC that confer auxin responsiveness (auxin-response element or AuxRe) have been reported [10,85,86]. In *P. xenovorans* LB400, a potential auxin-response element (TGTCAC) located upstream of the *ipdC* gene and the gene that encodes the transcriptional regulator from the AsnC family has been observed, suggesting that IAA may regulate their gene expression.

**Figure 4:**
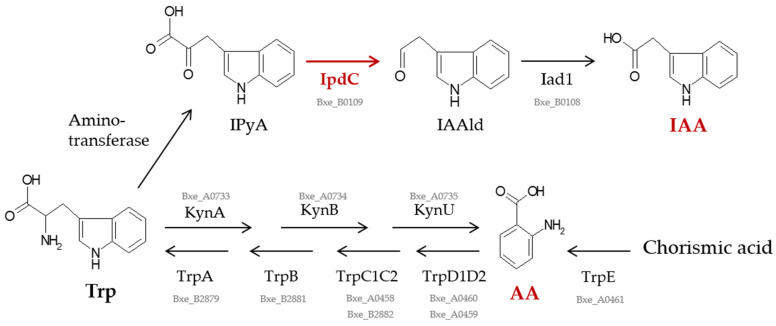
Proposed *P. xenovorans* LB400 biosynthetic pathways for the indole-3-acetic acid (IAA) and anthranilic acid (AA). Strain LB400 probably employs the IPyA pathway for IAA synthesis. The key substrates, metabolites, and products include anthranilic acid (AA), tryptophan (Trp), indole-3-pyruvic acid (IPyA), indole-3-acetaldehyde (IAAld), and indole-3-acetic acid (IAA). The enzymes involved in the pathway are an aminotransferase, indole-3-pyruvate decarboxylase (IpdC), and indole-3-acetaldehyde dehydrogenase (Iad1). Additionally, enzymes related to the metabolism of tryptophan to AA are tryptophan 2,3-dioxygenase (KynA), kynurenine formamidase (KynB), and kynureninase (KynU). Enzymes involved in the conversion of chorismate via AA to tryptophan are anthranilate synthase components I and II (TrpE and TrpD), indole-3-glycerolphosphate synthase/N(-5-phosphoribosyl) anthranilate isomerase (TrpC), and tryptophan synthase subunits β and α (TrpB and TrpA). Compounds and genes measured in this study are highlighted in red. The synthesis of the compounds IAA and AA (determined by HPLC) and the expression of the *ipdC* gene that encodes the enzyme indole-3-pyruvate decarboxylase (determined by RT-PCR). The genes encoding enzymes not highlighted are also present in the genome of strain LB400. The gene locus of the genes corresponding to strain LB400 are shown in grey.

In contrast, the *iaaH* gene from strain LB400, associated with the IAM pathway, showed no expression under the tested culture conditions (Figure 3c). This transcriptional analysis suggests the utilization of the IPyA pathway associated with PGPB for IAA biosynthesis in *P. xenovorans* LB400. Figure 4 shows the proposed biosynthetic pathways for IAA and AA production by strain LB400.

### 2.2. IAA Degradation

#### 2.2.1. *P. xenovorans* LB400 Possesses Genes for IAA Degradation

**Figure 5:**
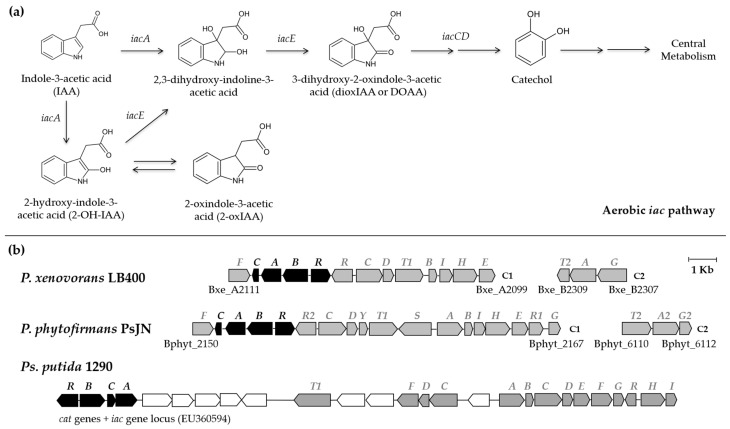
Indole-3-acetic acid (IAA) aerobic degradation pathway and gene clusters involved in IAA catabolism in *P. xenovorans* LB400, *P. phytofirmans* PsJN, and *Ps. putida* 1290. (**a**) IAA degradation pathway wherein the *iac* genes encoding the catabolic enzymes are indicated. The IAA aerobic degradation pathway was adapted from Laird et al. [18]. (**b**) The *iac* and *cat* genes involved in peripheral IAA pathway and central catechol pathway. The *iac* genes (grey) encode enzymes involved in the conversion of IAA to catechol, which is further metabolized by enzymes encoded by the *cat* genes (black). Gene sizes and intergenic regions are accurately represented to scale. C1 and C2 denote the major and minor chromosomes, respectively.

The proposed IAA degradation pathway of *P. xenovorans* LB400 wherein the *iac* genes encoding the catabolic enzymes is shown in Figure 5a. The bioinformatic analysis of the *iac* genes involved in IAA catabolism in *P. xenovorans* LB400 showed high identity with the genes of the catabolic pathway from *Paraburkholderia phytofirmans* PsJN and *Pseudomonas putida* 1290 [16,17]. LB400 structural proteins exhibited ≥ 40% amino acid identity with reported proteins of the IAA catabolic pathway (Table 3). The IAA catabolic pathway is also present in *Caballeronia glathei* DSM 50014, *Enterobacter soli* LF7, *Acinetobacter baumannii* ATCC 19606, *Aromatoleum evansii* KB 740, and *Aromatoleum aromaticum* EbN1 [18]. The transcriptional regulator encoded by the *P. xenovorans* LB400 *iacR* gene showed high identity with the regulator of the LysR type from *P. phytofirmans* PsJN (Table 3), while no significant similarity is found in the genome of strain LB400 with the transcriptional regulator belonging to the family MarR from *Ps. putida* 1290. In the LB400 genome, nine genes were contiguous in C1, and the other three were clustered in C2. Within the cluster associated to IAA degradation, the *cat* operon involved in catechol degradation was identified. The organization of these genes on *P. xenovorans* LB400 C1 is the *iacF*–*catCABR*–*iacRCDT1BIHE* cluster (Figure 5b). Additionally, the *iacT2AG* cluster is located on C2. This organization of genes related to IAA and catechol degradation is also observed in *P. phytofirmans* strain PsJN [17]. In *Ps. putida* 1290 (Figure 5), *A. baumanii* ATCC19606, and *E. soli* LF7, the *cat* genes are located close to the *iac* genes. This genomic organization highlights the relationship between *iac* and *cat* genes in IAA degradation, wherein the *iac* genes are involved in the conversion of IAA to catechol, which is further metabolized by enzymes encoded by the *cat* genes [18]. Genomic analyses in *Burkholderiales* including diverse members of the *Paraburkholderia* and *Burkholderia* genera showed the genes of the catabolic pathway of IAA to catechol [87].

#### 2.2.2. Degradation of IAA by *P. xenovorans* LB400

**Figure 6:**
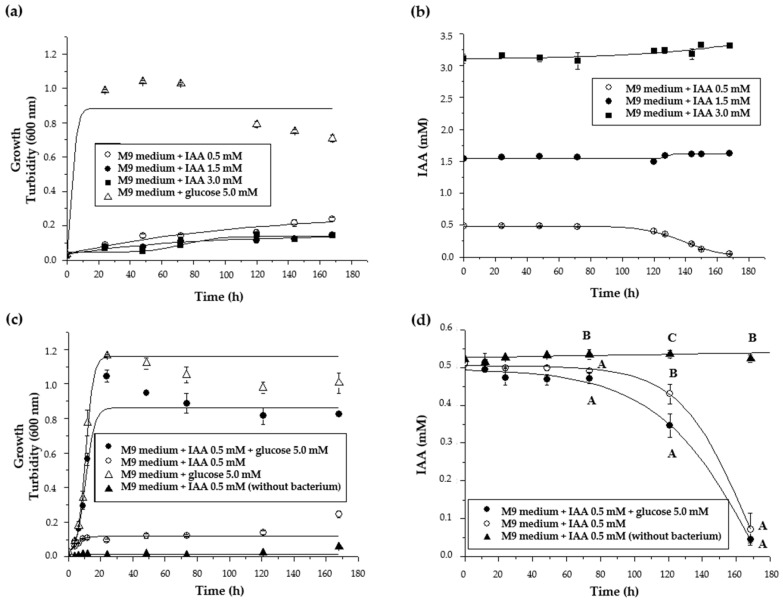
*P. xenovorans* LB400 growth on IAA and other carbon sources and IAA degradation. (**a**) LB400 growth on IAA (0.5, 1.5, and 3.0 mM). (**b**) Degradation of IAA (0.5, 1.5, and 3.0 mM). (**c**) LB400 growth on IAA (0.5 mM), IAA (0.5 mM) + glucose (5.0 mM), and glucose (5.0 mM). (**d**) Comparison of IAA (0.5 mM) degradation in co-culture with or without glucose (5.0 mM). Each value is a mean ± SD of three independent trials. Significant differences of the last three points of (**d**) were analyzed by one-way ANOVA followed by the LSD Fisher test. Means with different letters indicate significant differences (*p* ≤ 0.05).

The IAA degradation pathway in *P. xenovorans* LB400 was first evaluated through growth assays using IAA as the sole carbon and energy source. Growth and IAA degradation analyses were carried out using three IAA concentrations (0.5, 1.5, and 3.0 mM). Figure 6a shows that *P. xenovorans* LB400 growth on IAA is lower compared to growth on glucose (5 mM). *P. xenovorans* LB400 growth on IAA reached a turbidity (600 nm) of 0.25, while the growth with glucose reached a turbidity (600 nm) of 1.0. *P. xenovorans* LB400 was able to degrade lower IAA concentration (0.5 mM), but not higher IAA concentrations (1.5 and 3.0 mM) (Figure 6b). Strain LB400 started the degradation late (120 h), correlating with slight growth. Similar growth (turbidity (600 nm) ~0.3) of *Ps. putida* 1290 and *P. phytofirmans* PsJN have been reported, but with a 10-fold higher IAA concentration (5 mM) and after 20 and 60 h of growth, respectively [16,17]. To possibly enhance the growth and degradation of IAA, culture was performed with IAA (0.5 mM) plus glucose (5 mM) (Figure 6c); however, despite the higher growth on glucose, the IAA degradation did not increase (Figure 6d).

Interestingly, IAA exerted an inhibitory effect on *P. xenovorans* LB400 growth on glucose. *Lactobacillus*, *Saccharomyces cerevisiae*, and *Fusarium graminearum* showed an inhibition on their growth in the presence of IAA [9,88,89,90]. IAA concentrations of 1.14 and 5.7 mM exhibited growth inhibition in *Lactobacillus* sp. strain 11201, attributed to the accumulation of the metabolic product 3-methylindole (skatole) [91]. Skatole showed bacteriostatic effects in Gram-negative bacteria [92], whereas catechol and other dehydroxylated aromatic metabolites are highly toxic for bacteria [93]. Therefore, IAA or its degradation products could inhibit the growth of *P. xenovorans* LB400.

**Figure 7:**
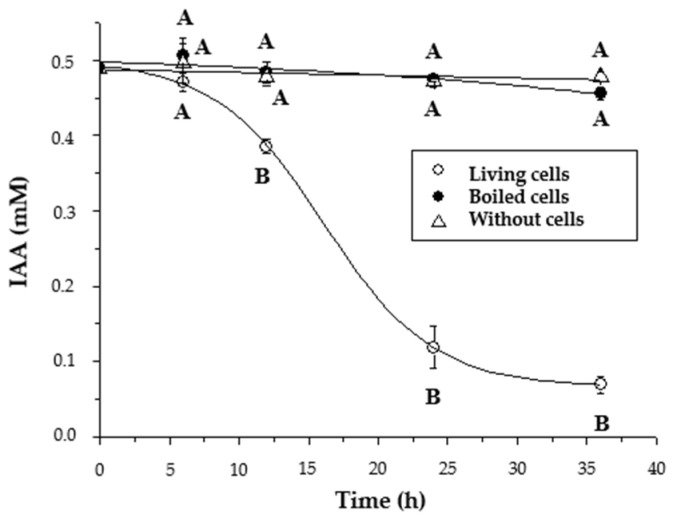
Degradation of IAA by *P. xenovorans* LB400 resting cells. This assay was performed with concentrated LB400 cells (turbidity 600 nm = 8.0) incubated in phosphate buffer (5.0 mM, pH 7.0) with IAA (0.5 mM). Each value is a mean ± SD of three independent assays. Significant differences were analyzed by one-way ANOVA followed by the LSD Fisher test. Means with different letters indicate significant differences (*p* ≤ 0.05).

To further study the degradation of IAA, *P. xenovorans* LB400 resting cells were incubated with IAA, resulting in almost complete degradation after 36 h (Figure 7). *P. xenovorans* LB400 exhibited the capability to degrade IAA and use it as sole carbon and energy source. However, this degradation might be more efficient under nutrient scarcity.

#### 2.2.3. *P. xenovorans* Expressed the *iacC* and *catA* Genes

**Figure 8:**
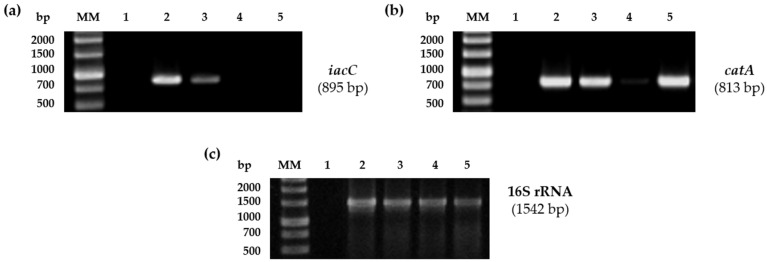
Expression of the *iacC* and *catA* genes in *P. xenovorans* LB400 cells incubated with different carbon sources. The expression was measured by RT-PCR. (**a**) Expression of the *iacC* gene. (**b**) Expression of the *catA* gene. (**c**) Expression of the 16S rRNA gene (reference gene). MM, molecular markers (UltraRanger 1 kb DNA ladder); 1, negative control; resting cells with 2, IAA (1 mM); 3, IAA (1 mM) + glucose (5 mM); 4, glucose (5 mM); 5, cells grown on salicylate (5 mM) + glucose (5 mM).

The expression of IAA catabolic genes in *P. xenovorans* LB400 was analyzed by assessing the expression of the *iacC* gene (Bxe_A2105), encoding the α subunit of the key dioxygenase in IAA catabolism, and the *catA* gene (Bxe_A2109), which encodes catechol 1,2-dioxygenase. The expression of the *iacC* and *catA* genes was measured by RT-PCR analysis in LB400 cells incubated with IAA and glucose (Figure 8). Growth in the presence of salicylate plus glucose was incorporated as a positive control, as salicylate is degraded via the central catechol pathway [21] The expression of the *iacC* gene was observed in resting cells incubated with IAA, and IAA plus glucose, but not with glucose or salicylate plus glucose (Figure 8a). On the other hand, the expression of the *catA* gene was observed in resting cells with IAA, IAA plus glucose, and salicylate plus glucose (Figure 8b). The expression of the *catA* gene in cells incubated with glucose might be attributed to the degradation of compounds such as tryptophan [21]. The expression of diverse *iac* genes and their convergence in the catechol degradation pathway has been described in *Ps. putida* 1290, *Acinetobacter baumanii* ATCC 19606, *P. phytofirmans* PsJN, *Enterobacter soli* LF7, and *Caballeronia glathei* DSM50014 [17,18,94,95,96]. The expression of the *iacC* gene is probably associated with the transformation of the metabolic intermediate 3-hydroxy-2-oxoindole 3-acetic acid (DOAA) into catechol [18] (Figure 5a). The expression of *iacC* and *catA* genes in *P. xenovorans* LB400 may suggest that this strain has an active catabolic pathway that converts IAA into catechol [18]. The IAA peripheral pathway is probably connected to the central catechol pathway, which is involved in the degradation of various compounds such as biphenyl, benzonitrile, benzaldehyde, benzamide, benzoate, mandelate, salicylate, anthranilate, and tryptophan [21].

### 2.3. Promotion of Nicotiana Tabacum Plant Growth by P. xenovorans LB400

**Figure 9:**
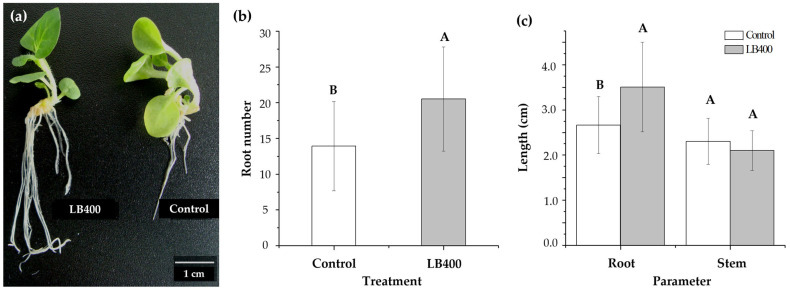
Growth promotion of *P. xenovorans* LB400 on *Nicotiana tabacum* seedlings. (**a**) Representative *N. tabacum* seedlings post-treatment with strain LB400 and water, respectively. (**b**) Effects of strain LB400 on the number of roots in *N. tabacum*. (**c**) Effects of strain LB400 on root and stem length in *N. tabacum*. Each value is a mean ± SD of 12 biologically independent replicates. Significant differences were analyzed by one-way ANOVA followed by the LSD Fisher test. Means with different letters indicate significant differences (*p* ≤ 0.05).

The growth promotion of *P. xenovorans* LB400 on the plant *N. tabacum* was studied. Strain LB400 (10^7^ CFU mL^−1^) was applied on two-week-old *Nicotiana tabacum* seedlings by inoculating the bacterium into the first root. Plant analyses were conducted two weeks after strain LB400 application (Figure 9). Inoculation with strain LB400 significantly increased both the number and length of roots in *N. tabacum* seedlings. Specifically, the root number per seedling increased from 14 roots in the water-treated group to 21 roots per seedling in the strain LB400-treated plants (Figure 9b). In addition, strain LB400-treated seedlings exhibited longer roots (3.5 cm) compared to the water-treated group (2.7 cm) (Figure 9c). However, strain LB400 did not affect the stem length (Figure 9c). The plant growth-promoting effect of *P. xenovorans* LB400 may be associated with its IAA synthesis. Tryptophan, a precursor for IAA synthesis, is released by seeds, seedlings, and roots, allowing PGPB to enhance plant growth [97,98]. The growth-promoting effect of strain LB400 may arise from IAA production, directly stimulating plant cell elongation. The *ipdC* gene knock-out mutants of *Azospirillum brasilense* SM and *Bacillus thuringiensis* RZ2MS9 showed reduced growth-promoting effects on sorghum seeds and maize [39,82].

Plant growth-promotion by *P. xenovorans* strains has been reported [35,99]. Recently, the ability of strain LB400 to promote in vitro *Arabidopsis thaliana* growth has been described [58]. Other properties may be involved in plant growth-promotion by strain LB400, such as the nitrogen-fixing activity, ACC deaminase activity, the synthesis of siderophores, volatile organic compounds, extracellular polymeric substances, and polyhydroxyalcanoates (PHAs) [35,58,100,101,102]. The role of PHAs in plant–bacteria interactions is poorly understood; however, studies with the bacterium *Herbaspirillum seropedicae* suggest that PHAs metabolism favors the expression of traits that promote plant growth [103]. On the other hand, the presence of genes related to different types of stress in bacteria could be associated to a robust adaptive response [104,105,106]. The oxidative stress protection response improves the plant growth promotion activity of bacteria under abiotic stress conditions [107,108]. In addition to its capability to degrade an unusually wide range of pollutants and aromatic compounds [21,22,23,24,27,28,29,30,106], *P. xenovorans* LB400 possesses an extraordinaire oxidative stress response [25,31,106,109] and the capability to synthesize PHAs from different carbon sources [100,102,110]. Its plant growth promotion activities reported in this study increase the potential biotechnological applications of the versatile bacterium *P. xenovorans* LB400 and highlighted the importance of opening new doors for the characterization of the potential biotechnological and ecosystemic services of a specific native bacterial strain.

## 3. Materials and Methods

### 3.1. Bacterial Strains

In this study, the bacterial strains *P. xenovorans* LB400, a PCB-degrading bacterium that was isolated from PCB-polluted soil in New York State [21], and *Ps. protegens* CHA0, a plant growth-promoting bacterium capable of synthesizing IAA and isolated from tobacco roots in Switzerland [71,111], were used. These strains were obtained from the culture collections of the Molecular Microbiology and Environmental Biotechnology Laboratory, Universidad Técnica Federico Santa María, Valparaíso, Chile.

### 3.2. Bioinformatic Analysis

Genes related to IAA synthesis and degradation were searched in the LB400 genome. The protein amino acid sequences were obtained through the EMBL Nucleotide Sequence Database (http://www.ebi.ac.uk/embl/; accessed on 15 September 2024) and NCBI (http://www.ncbi.nlm.nih.gov/; accessed on 15 September 2024) servers. The sequences found were used as a template in the bioinformatic analysis using the complete genome sequence of *P. xenovorans* LB400 (https://www.genome.jp/kegg-bin/show_organism?org=bxe; accessed on 15 September 2024). Using the NCBI BLAST tool, specifically blastp, for the alignment of amino acid sequences, ≥30% amino acid identity was used for the identification of proteins of strain LB400 [106].

### 3.3. Synthesis of IAA

For IAA synthesis, *P. xenovorans* LB400 was cultured at 30 °C in mineral M9 medium supplemented with a trace solution [27] and glucose (30 mM), and in the absence and presence of *DL*-tryptophan (10 mM), and LB medium containing *DL*-tryptophan (10 mM) at 30 °C and 180 rpm. Samples were taken at 0, 24, 48, and 72 h. Comparative IAA production assays were performed with *P. xenovorans* strain LB400 and *Ps. protegens* CHA0. Both strains were cultured in LB, KB, and YM media with the addition of *DL*-tryptophan (5 mM) at 30 °C and 180 rpm. To assess bacterial growth, colony-forming units (CFUs) were determined. Aliquots extracted from the cultures were appropriately diluted and plated on LB agar medium for strain LB400 and on KB agar medium for strain CHA0. The CFUs per milliliter values were calculated as the mean ± SD, based on at least three independent experiments. Aliquots of 1 mL were taken and stored at −30 °C until further analysis.

### 3.4. Degradation of IAA

For IAA degradation, *P. xenovorans* LB400 was cultured in LB liquid medium at 30 °C at 180 rpm for 12 h, reaching a turbidity at 600 nm > 1. An inoculum of this culture was added to M9 minimal medium in the presence of IAA (0.5, 1.5, and 3.0 mM) as the sole carbon and energy source, and incubated for 7 days (in triplicate). As a growth control, strain LB400 was grown in glucose (5 mM) (in triplicate). In a second assay, a co-culture with glucose (5 mM) and IAA (0.5 mM) was incubated for 7 days (in triplicate), comparing it with growth on glucose and IAA as sole carbon and energy sources. To accelerate the IAA degradation, *P. xenovorans* resting cells assays [27] were performed. A first preculture (20 mL) was carried out in M9 medium with glucose (5 mM), which was incubated at 30 °C at 180 rpm for 24 h. The next day, this preculture was used as inoculum for a 200 mL culture. This second culture was carried out in M9 medium with glucose (5 mM) and IAA (0.5 mM), incubating it at 30 °C at 180 rpm for 48 h. Then, this culture was washed twice with sodium phosphate buffer (5 mM; pH 7) and centrifuged at 3500× *g* for 15 min at 4 °C. The pellet was then resuspended in sodium phosphate buffer (5 mM; pH 7) in a final volume of 8 mL, reaching a turbidity (600 nm) of 8.0. In this assay, two controls were performed, one with dead cells (autoclaved culture, 121 °C for 30 min) and one without cells. Only IAA (0.5 mM) was added to these three treatments, and they were incubated at a temperature of 30 °C at 180 rpm for 36 h. Samples were taken at 0, 6, 12, 24, and 36 h. The assays were performed in triplicate. Aliquots of 600 µL were taken and stored at −30 °C until further analysis.

### 3.5. Quantification of IAA and AA

The aliquots taken from the synthesis and degradation assays were centrifugated at 19,283× *g* for 2 min at 4 °C and the resulting cell-free supernatants underwent analysis through reverse-phase chromatography. Therefore, a Jasco liquid chromatograph, equipped with a diode array detector and a Whatman C-18 reversed phase column (5 µm; 4.6 × 100 mm), was used. Quantification of IAA was conducted following the protocol outlined by Lee et al. [6] with some modifications. The aqueous mobile phase consisted of 70% acetic acid (1.1%) and 30% acetonitrile, with a flow rate at 1 mL min^−1^. IAA was monitored at wavelengths of 221 and 280 nm, and its retention time under these conditions was 3.1 min. Anthranilic acid (AA) was detected at 221 nm with a retention time of 2.6 min. For the quantification of both IAA (0 to 2.0 mM) and AA (0 to 2.0 mM), calibration curves using commercial authentic standards were performed.

### 3.6. Isolation of RNA and RT-PCR

Total RNA extractions from LB400 cells were performed using the RNeasy mini kit (Qiagen, Hilden, Germany), following the manufacturer’s guidelines. To eliminate any residual DNA, DNase I treatment was carried out using the RNase-Free DNase Set (Qiagen, Hilden, Germany). The concentration of RNA was quantified using a NanoDrop 1000 spectrophotometer (Thermo Scientific, Lafayette, LA, USA), and RNA integrity was verified through 1% agarose gel electrophoresis. For RT-PCR, 1000 ng of total RNA was transcribed using the Verso cDNA kit (Thermo Scientific, Lafayette, LA, USA) according to the manufacturer’s instructions. The subsequent PCR was performed with GoTaq Green Master Mix (Promega, Madison, WI, USA), utilizing sequence-specific primers designed in this study for the *ipdC* (Bxe_B0109), *iaaH* (Bxe_B1011), *iacC* (Bxe_A2105), and *catA* (Bxe_A2109) genes.

### 3.7. RT-PCR of Genes Associated to IAA Metabolism

For the study of IAA synthesis, *ipdC* and *iaaH* genes were analyzed. The RNA extractions were performed after 24 and 48 h of cultures of strain LB400 under the following conditions: (i) M9 medium + glucose (30 mM), (ii) M9 medium + glucose (30 mM) + tryptophan (10 mM), and (iii) LB medium + tryptophan (10 mM). The Bxe_B0109 gene (*ipdC*) was amplified using the primers IPDC-f and IPDC-r (Table 4), whereas the Bxe_B1011 gene (*iaaH*) was amplified using the primers IAAH-f and IAAH-r (Table 4).

For the study of the IAA degradation pathway, *iacC* and *catA* genes were analyzed. The RNA extractions were performed after 24 h of cultures of strain LB400 under the following conditions: (i) resting cells + IAA (1 mM), (ii) resting cells + IAA (1 mM) + glucose (5 mM), (iii) resting cells + glucose (5 mM), and (iv) cells grown in salicylate (5 mM) + glucose (5 mM). The Bxe_A2105 gene (*iacC*) was amplified using the primers IACC-f and IACC-r (Table 4) and the Bxe_A2109 gene (*catA*) was amplified using the primers CATA-f and CATA-r (Table 4).

The PCR program for the amplification of *ipdC*, *iaaH*, *iacC*, and *catA* was initial denaturation at 94 °C for 5 min, following 30 cycles of 94 °C for 1 min, 58 °C for 1 min, and 72 °C for 1 min, and with a final elongation at 72 °C for 7 min. Each PCR assay included negative controls, and reproducibility was assessed through at least two independent RT-PCR reactions for each condition. For the study of the IAA synthesis pathway, different amplification cycles (30, 25, 20, and 15) were used. The primers designed in this study were tested through amplification of LB400 genomic DNA (Figure S1). The amplification of the 16S rRNA gene was used as a control for DNA contamination (Figures S2 and S3) and gene expression (Figure 3 and Figure 8), with the primers 27f and 1492r [27]. The PCR program used was an initial denaturation at 94 °C for 5 min, following 30 cycles of 94 °C for 1 min, 55 °C for 1 min, and 72 °C for 2 min, and with a final elongation at 72 °C for 7 min.

### 3.8. Nicotiana tabacum Growth-Promoting Assay

*Nicotiana tabacum* seeds were subjected to a sterilization process involving a brief immersion in 70% ethanol for 30 s, followed by exposure to 1% sodium hypochlorite for 10 min, and subsequently rinsed twice with sterile distilled water. The sterilized seeds were germinated on Murashige–Skoog (MS) agar plates [112], containing macro- and micronutrients, 3% sucrose, and 8.0 g L^−1^ agar, during a two-week period. The germination process occurred under a photoperiod of 14 h light and 10 h dark at a temperature of 25 °C. For the experimental setup, 15 cm test tubes were utilized, each containing MS agar slant (5 mL) to provide an ample surface for seedling growth. Two distinct treatments were implemented, one with sterile distilled water and the other with 10^7^ CFU mL^−1^ of LB400 cells. In each tube (12 tubes per treatment), 100 µL of sterile distilled water or LB400 cells suspension was added. *Nicotiana tabacum* seedlings were then carefully inserted into the agar, and the tubes were sealed with parafilm. The experiment continued for two weeks under a photoperiod of 14 h light and 10 h dark at a temperature of 25 °C.

### 3.9. Statistical Analysis

In the assessment of IAA synthesis, AA synthesis, IAA degradation, and plant growth-promoting assays, the mean and standard deviation values were based on a minimum of three independent assays. Comparative analyses were subjected to one-way ANOVA. After the ANOVA analysis, significant differences (*p* ≤ 0.05) between treatments were further examined utilizing the LSD Fisher mean difference test.

## 4. Conclusions

This study indicates that *P. xenovorans* LB400 has functional pathways for the synthesis and degradation of the phytohormone IAA. Strain LB400 cultured with the precursor tryptophan and another carbon source synthesized IAA. Under these conditions, strain LB400 also presented the ability to synthesize and accumulate anthranilic acid. *P. xenovorans* LB400 expressed the *ipdC* gene that codes for the enzyme indole-3-pyruvate decarboxylase, suggesting the IPyA pathway for the biosynthesis of IAA, a route that has been described mainly for plant growth-promoting bacteria. *P. xenovorans* LB400 showed the capability to degrade IAA, and to use this substrate as the sole carbon and energy source for its growth. During the incubation in the presence of IAA, strain LB400 expressed the *iacC* gene, which encodes the α subunit of the key dioxygenase in the catabolism of IAA, suggesting that it uses a specific catabolic route. In addition, strain LB400 expressed the *catA* gene, which encodes catechol 1,2-dioxygenase, suggesting that the peripheral IAA catabolic pathway converges into the catechol central pathway. However, additional assays are required to confirm these IAA synthesis and degradation pathways. Notably, strain LB400 promoted growth of *Nicotiana tabacum* seedlings, which may be associated with its capability to synthesize IAA; therefore, this versatile bacterium has been classified as a PGPB.

## Figures and Tables

**Table 1:** Proteins associated to the IPyA and IAM pathways for IAA biosynthesis in *P. xenovorans* LB400.

Pathway	Gene	ORF	aa	Related Gene Products			
				Protein	Function	Organism (Accession Number)	Identity (%)
IPyA	*ipdC*	Bxe_B0109	550	IpdC	Indole-3-pyruvate decarboxylase	*Pantoea agglomerans* 299R (WP_003848906)	229/539 (42)
	*iad1*	Bxe_B0108	497	Iad1	Indole-3-acetaldehyde dehydrogenase	*Ustilago maydis* FB1 (AAC49575)	176/481 (37)
IAM	*iaaM*	Bxe_C1245	575	IaaM	Tryptophan-2- monooxygenase	*Agrobacterium fabrum* C58 (AAD30489)	212/557 (38)
	*iaaH*	Bxe_B1011	484	IaaH	Indole-3-acetamide hydrolase	*Agrobacterium fabrum* C58 (AAD30488)	200/466 (43)

Abbreviations: IPyA: indole-3-pyruvate; IAM: indole-3-acetamide; IAA: indole-3-acetic acid; ORF: open reading frame; aa: amino acid.

**Table 2:** IAA and AA biosynthesis of *P. xenovorans* LB400 and *Ps. protegens* CHA0 grown in different culture media in the presence of tryptophan (5 mM) during 48 h.

Bacterial Strain	Medium	Average IAA (mM) Synthesis (±SD)	Average AA (mM) Synthesis (±SD)	Average log (CFUs mL^−1^) (±SD)
*P. xenovorans* LB400	LB	0.193 (±0.012)	0.141 (±0.034)	5.349 (±0.494)
	YM	0.404 (±0.010)	0.477 (±0.016)	7.455 (±0.160)
	KB	0.167 (±0.006)	2.013 (±0.171)	6.301 (±0.426)
*Ps. protegens* CHA0	LB	0	0	7.331 (±0.142)
	YM	0.124 (±0.009)	0.095 (±0.020)	6.540 (±0.088)
	KB	0	0.046 (±0.031)	7.752 (±0.016)

Abbreviations: IAA: indole-3-acetic acid; AA: anthranilic acid; CFUs: colony-forming units; SD: standard deviation.

**Table 3:** Proteins associated to IAA degradation in *P. xenovorans* LB400.

Gene	ORF	aa	Related Gene Products		
			Protein	Function	Identity (%)
*iacA*	Bxe_B2308	405	IacA	Acyl-CoA dehydrogenase flavoprotein	187/374 (50) *
*iacB*	Bxe_A2102	125	IacB	Hypothetical conserved protein	65/119 (55) *
*iacC*	Bxe_A2105	427	IacC	Aromatic ring hydroxylation dioxygenase, α subunit	256/418 (61) *
*iacD*	Bxe_A2104	166	IacD	Aromatic ring hydroxylation dioxygenase, β subunit	69/147 (47) *
*iacE*	Bxe_A2099	247	IacE	Short-chain dehydrogenase	129/245 (53) *
*iacF*	Bxe_A2111	325	IacF	Ferredoxin reductase	131/328 (40) *
*iacG*	Bxe_B2309	183	IacG	Flavin reductase domain protein	72/154 (47) *
*iacH*	Bxe_A2100	374	IacH	Amidase	193/376 (51) *
*iacI*	Bxe_A2101	180	IacI	Hypothetical conserved protein	67/151 (44) *
*iacT1*	Bxe_A2103	438	IacT1	Transporter major facilitator superfamily (MFS) (DOAA transporter)	420/438 (96) **
*iacT2*	Bxe_B2307	452	IacT2	Transporter major facilitator superfamily (MFS) (DOAA transporter)	221/436 (51) **
*iacR*	Bxe_A2106	317	IacR	Transcriptional regulator LysR family	301/315 (96) **

* Compared to the protein of *Pseudomonas putida* 1290 [16]. ** Compared to the protein of *Paraburkholderia phytofirmans* PsJN [17]. Abbreviations: IAA: indole-3-acetic acid; ORF: open reading frame; aa: amino acid; DOAA: 3-dihydroxy-2-oxoindole-3-acetic acid.

**Table 4:** Primers used in this study.

Primer	Gene	Sequence (5′–3′)	Reference
IPDC-f	*ipdC*	CATCGTTGAAGCCCTTGCGT	This study
IPDC-r		TCGCCGATGAACAGCAAATGG	This study
IAAH-f	*iaaH*	TTCGTGCCGAAGACCAATGC	This study
IAAH-r		TCGTAACGCTCGCCGAAGATA	This study
IACC-f	*iacC*	AGCGGAACTCGAACGCATTT	This study
IACC-r		TCGCCGATGAACAGCAAATGG	This study
CATA-f	*catA*	TTGCTCCAGAAGATCAACGA	This study
CATA-r		GAAATCGATCAACGCGAAAT	This study
27f	16S rRNA	AGAGTTTGATCMTGGCTCAG	[27]
1492r		TACGGYTACCTTGTTACGACTT	[27]

## Data Availability

Data are contained within the article and Supplementary Materials.

## Supplementary Materials

The following supporting information can be downloaded at: https://www.mdpi.com/article/10.3390/plants13243533/s1, Figure S1: Functionality test of the primers designed in this study for the *ipdC*, *iaaH*, *iacC*, and *catA* genes; Figure S2: Amplification of 16S rRNA gene of IAA synthesis RNA samples for control of DNA contamination; Figure S3: Amplification of 16S rRNA gene of IAA degradation RNA samples for control of DNA contamination.
