# Supplementary material for: Synthesis and Degradation of the Phytohormone Indole-3-Acetic Acid by the Versatile Bacterium Paraburkholderia xenovorans LB400 and Its Growth Promotion of Nicotiana tabacum Plant
Source: Plants (Basel). 2024 Dec 18;13(24):3533. doi: 10.3390/plants13243533 (PMC11676955; doi:10.3390/plants13243533)
Supplement: Supplementary file 1 [file plants-13-03533-s001.zip › plants-3295564-supplementary.pdf]

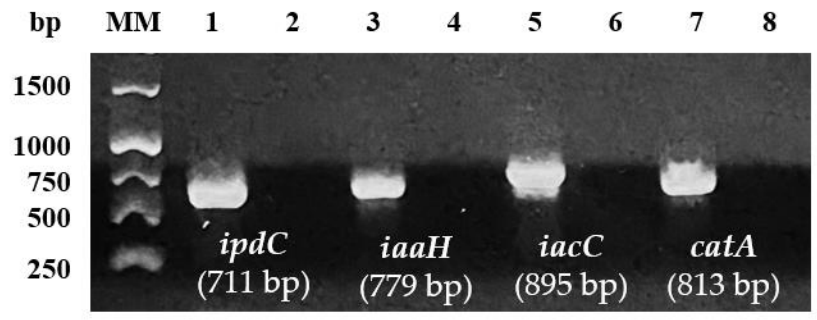

Figure S1. Functionality test of the primers designed in this study for the *ipdC*, *iaaH*, *iacC*, and *catA* genes. MM, molecular markers (UltraRanger 1 kb DNA ladder); *ipdC* gene: 1, LB400 genomic DNA; 2, negative control (without genomic DNA); *iaaH* gene: 3, LB400 genomic DNA; 4, negative control; *iacC* gene: 5, LB400 genomic DNA; 6, negative control; *catA* gene: 7, LB400 genomic DNA; 8, negative control.

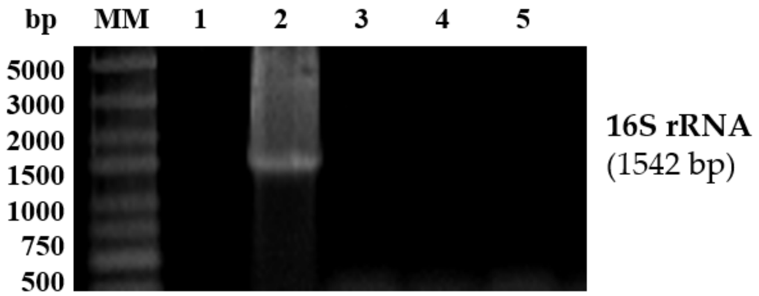

Figure S2. Amplification of 16S rRNA gene from IAA synthesis RNA samples for control of DNA contamination. MM, molecular markers (GeneRuler Express DNA Ladder); 1, negative control (without genomic DNA); 2, LB400 genomic DNA (positive control); RNA samples from cells grown in: 3, M9 medium supplemented with glucose (30 mM); 4, M9 medium supplemented with glucose (30 mM) and tryptophan (10 mM); 5, LB medium supplemented with tryptophan (10 mM).

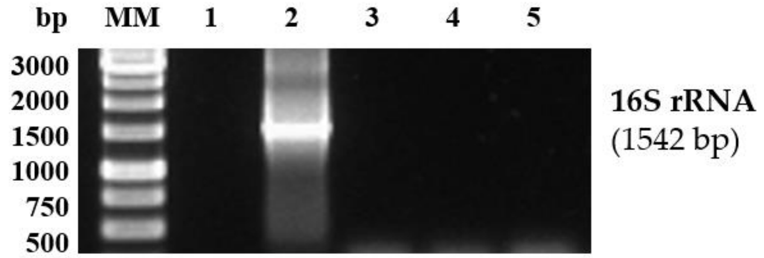

Figure S3. Amplification of 16S rRNA gene from IAA degradation RNA samples for control of DNA contamination. MM, molecular markers (UltraRanger 1 kb DNA ladder); 1, negative control (without genomic DNA); 2, LB400 genomic DNA (positive control); RNA samples of LB400 resting cells with: 2, IAA (1 mM); 3, IAA (1 mM) + glucose (5 mM); 4, glucose (5 mM); 5, cells grown on salicylate (5 mM) + glucose (5 mM).
